# Public health research systems in the European union

**DOI:** 10.1186/1478-4505-9-38

**Published:** 2011-10-04

**Authors:** Cláudia Conceição, Mark McCarthy

**Affiliations:** 1 AGO, Associação para o Desenvolvimento e Cooperação Garcia de Orta, Portugal; 2 Department of Epidemiology and Public Health, University College London, UK

## Abstract

**Background:**

Strengthening health research is an important objective for international health organisations, but there has been less attention to support for health research in Europe. We describe the public-health (population and organisational level) research systems in the 27 European Union countries.

**Methods:**

We developed a typology for describing health research structures based on funding streams and strategies. We drew data from internet sources and asked country informants to review these for consistency and completeness. The structures were described as organograms and narratives in country profiles for each of the 27 EU member states. National public-health research structures included public and independent funding organisations, 'mixed' institutions (which receive funds, and both use and allocate them) and provider institutions.

**Results:**

Most health research is funded through ministries of science or science councils (and sometimes foundations), while parliaments and regions may also contribute. National institutes of public health are usually funded by ministries of health. Many national research organisations both determine research programmes and undertake health research, but there is a move towards public-health sciences within the universities, and a transition from internal grants to competitive funding. Of 27 national research strategies, 17 referred to health and 11 to public health themes. Although all countries had strategies for public health itself, we found little coherence in public-health research programmes. The European Commission has country contact points for both EU research and health programmes, but they do not coordinate with national health-research programmes.

**Conclusions:**

Public-health research is broadly distributed across programmes in EU countries. Better understanding of research structures, programmes and results would improve recognition for public health in Europe, and contribute to practice. EU ministries of health should give greater attention to national public-health research strategies and programmes, and the European Union and the World Health Organisation can provide coordination and support.

## Background

The systems providing knowledge for evidence-based practice deserve research attention. The World Health Report in 2012 on Research for Health will provide "new ideas, innovative thinking, and pragmatic advice for member states on how to strengthen their own health research systems" [1]. In 2011, the European Commission has consulted on future funding for research in the European Union [2]. We have made a description and comparison of the systems for public-health research in the 27 countries of the European Union in support of these two initiatives.

A health research system has been defined as "the people, institutions, and activities whose primary purpose in relation to research is to generate high-quality knowledge that can be used to promote, restore, and/or maintain the health status of populations" [3]. Following the Report of the Commission on Health Research for Development [4], ministerial conferences were held, in Mexico in 2004 and Mali in 2008, led by the Global Forum for Health Research [5]. Governments from European countries (and also the European Union) attended both conferences, and the WHO European Region was involved in preparations for the Mali conference [6]. The WHO European Region is one of six WHO Regions, with more than a quarter (53) of all member states. In the western half of the region, the European Union (EU) includes 27 member states, with Norway, Iceland, Lichtenstein and Switzerland also closely allied through bilateral agreements, together representing more than 500 million people. While the majority of EU countries are high-income economies by World Bank measures [7], five countries (Bulgaria, Latvia, Lithuania, Poland and Romania) are middle income. There are also economic gradients both across and within the EU. The EU Structural and Cohesion Funds, a quarter of the EU's total budget, are directed towards all 12 new member states in eastern and southern areas, and also to regions economically below the average in almost all member states.

All EU member states have national research programmes [8] and health research systems are situated between the broader systems for research and health respectively [3]. EU countries also collaborate internationally in health research as donors, both bilaterally and together in funding through the European Commission [9]. The EU gives direct support to its member states currently through the Seventh Framework Research Programme (€50 bn 2007-2013), and also more through the Structural Funds (€84 bn 2007-2010) [10]. There is discussion and consultation on the funding and substance for the research and innovation programme from 2014 onwards [11].

As WHO members, the EU member states have approved the WHO Research for Health Strategy of the 63rd Regional Health Assembly [12] through their ministries of health. The European Commission's Directorate-General for Health and Consumers Directorate is responsible for health issues within the EU [13], with the main focus on public health (including protection), food safety and animal health, while under the EU Treaty [14], the European Commission's Directorate-General for Research is responsible for research across all areas of EU policy.

Life sciences and biomedical research have been an important part of the EU research programmes, second only in funding to information technology [15]. However, the EU policy responsibilities for public health have been less well represented in the research programmes. A first study of public health research in Europe, *SPHERE *(2005-2007) [16], drew together different European perspectives, from research organisations, civil society organisations and European institutions, and made a survey of national ministries of health and ministries of science to record their perceptions on public health research [15,17,18]. *SPHERE *also undertook bibliometric analysis of public health research publications across 6 areas: health services research, environmental health, infections disease control, health promotion, genetic epidemiology and health management [19-24]. Among the findings was a substantial geographical disparity in numbers of publications, from strong levels in Scandinavia and the UK to the lowest levels in the 12 EU new member states [25,26].

To investigate this disparity, in 2009 STEPS (Strengthening Engagement in Public Health Research) was funded by the European Commission's Science in Society programme [27]. STEPS has engaged civil society organisations in the 12 EU new member states in debate with ministries of health on support for public health research, and also to describe public-health research systems and programmes - how is Public Health research funded, which institutions undertake it, and what is supported - across the full 27 EU countries. Following the guidelines for reporting on observational health research, identified by the Equator Network [28,29], we present findings from this multi-country comparative study.

## Methods

The process to describe public-health research systems and programmes in individual EU countries was undertaken in two phases. While partners in STEPS were identifying contacts in the 12 EU new member states (Bulgaria, Cyprus, Czech Republic, Estonia, Hungary, Latvia, Lithuania, Malta, Poland, Romania, Slovakia, Slovenia) as part of its activity to hold country workshops on public health research, we worked initially with the former 15 EU countries (Austria, Belgium, Denmark, Finland, France, Germany, Greece, Ireland, Italy, Luxembourg, Netherlands, Portugal, Spain, Sweden and the United Kingdom). For each of these countries, we drew together information within a structured template.

Our experience in *SPHERE *had shown the limitations of asking ministry officials, who have many demands on their time, for information outside their normal areas of work. We therefore started from the profiles we had collected for *SPHERE *[30], and supplemented this with information on the web sites for ministries and official agencies (using Google translate for greater detail). We also reviewed information on the web sites of member organisations of several European coordinating groups, including EUnetHTA (organizations producing or contributing to health technology assessment) [31], EUROHORCS (European Heads of Research Councils) [32], IANPHI (International Association of National Public Health Institutes) [33], EuSANH (European Science Advisory Network for Health, a network of national public health boards) [34], and ASPHER (Association of Schools of Public Health in the European Region) [35]. We did not include, unless a relevant part of the mission was research, organisations with functions of environmental protection, radiation protection, pharmaco-vigilance or veterinary services/animal health.

We developed a template for recording country information, making revisions as information became available. The EU website ERAWATCH [8] (the European Commission's information platform on European, national and regional research systems and policies) includes reports from informants in each member states for the whole field of research. While individual countries have unique organisations and names, national structures can be considered similar from the perspective of funding flows - the organisations that disburse funds and commission research, and those that receive funds and perform research. We developed a model organogram reflecting different organisations and flows, also distinguishing competitive grant funding from direct allocations and subsidies. The organogram forms the first section of each Country Profile, with subsequent sections describing the structures and contents of research [36].

For each country profile, when we had completed a first draft, we sent it to an informant who returned the script with comments, amendments, corrections, and improvements. The manuscript was then revised iteratively. We gained our informants first from the respondents to *SPHERE *at the ministries of health or of science, or if they could no longer assist then from another person indicated by them, through our internet search or our personal contacts. The first contacts were established between mid May and mid June 2010. For these 15 countries, we sent 142 emails out (range 5-17), and received 65 back (range 1-11) (Table 1). In 10 of the 15 countries, informant comments were received from ministries of health or governmental organizations, and for the other countries the key informants were mainly related to Universities (Table 2).

**Table 1:** Date of first contact, of reception of comments, number of emails sent and received, by country

Country	Date of first contact	Date of initial replies	Number of emails sent	Number of emails received
Austria	9 June 2010	26 June 2010	5	2

Belgium	24 May 2010	31 May 2010	3	1

Denmark	24 May 2010	7 October 2010	17	11

Finland	24 May 2010	24 June 2010	5	3

France	24 May 2010	30 August 2010	13	3

Germany	8 June 2010	17 August 2010	14	8

Greece	8 June 2010	2 August 2010	7	5

Ireland	24 May 2010	16 July 2010	10	4

Italy	11 June 2010	1 September 2010	10	3

Luxembourg	24 May 2010	--	4	--

Netherlands	14 June 2010	10 August 2010	7	2

Portugal	24 May 2010	16 June 2010	8	3

Spain	8 June 2010	19 January 2011	16	8

Sweden	24 May 2010	5 July 2010	6	2

UK	8 June 2010	24 Sept. 2010	17	8

**Table 2:** Organisation of one or more final informants/reviewers by country

Country	Organisation of final key informants/reviewers
Austria	Ministry of health

Belgium	Ministry of health

Bulgaria	State institute (Ministry of health) and University

Cyprus	University

Czech Republic	University, Civil society organisation

Denmark	University

Estonia	Civil society organisation, University

Finland	State institute (Ministry of health) and Ministry of health

France	Health research agency (Ministry of health and Ministry of science)

Germany	University

Greece	University

Hungary	Ministry of health

Ireland	Ministry of health and Health research agency (Ministry of health)

Italy	Ministry of health

Latvia	University

Lithuania	Civil society organisation, University

Luxembourg	--

Malta	Civil Society Organisation, University

Netherlands	Ministry of health

Poland	University, University

Portugal	State institute (Ministry of health)

Romania	University

Slovakia	Civil society organisation, University

Slovenia	State institute (Ministry of health)

Spain	State institute (Ministry of health)

Sweden	Ministry of health

UK	University, University

The related ministries are shown in round brackets for the State institutes and Health research agencies.

In March and April 2010, workshops were held in each of the 12 EU new member states countries that were directly partners in STEPS, bringing together national public health associations, other civil society organizations, universities/researchers and ministries of health [37]. The workshops each included a description of the national research structures, although using different approaches. As for the first 15 countries, we made initial assessments of the research structures from internet sources, supplemented this with observations from country visits, and with each partner after the workshop we drew the information together in the period up to September 2010.

The template was developed with the following parts: Introduction, Organogram, Research Commissioners, Research Performers, Research Strategies, Programmes and Calls, and European Contacts. The Introduction included a brief presentation of the project, objectives of country profiles and methods to gather information and a brief definition was presented: 'Public-health research' includes all health research at population, organisation and system level broadly relevant to health and health-care policy and practice. It excludes clinical and laboratory (biomedical) research. The word concept of 'performers' is commonly used in describing research structures [8,38], while 'providers' is used in describing health care.

The country profiles were each further reviewed by the authors, and revised to ensure completeness and standardization of approach. They were placed on-line on the project website http://www.steps-ph.eu/country-health-research-profiles/ at the end of October 2010, and further review and additional comments led to updated information available in September 2011.

## Findings

### Organograms

**Figure 1:**
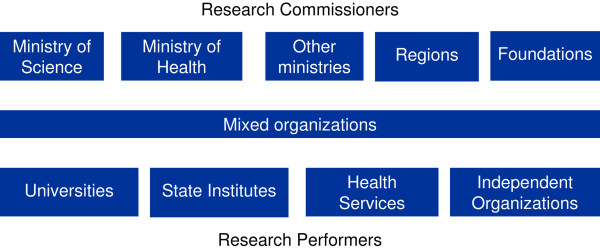
**Country profiles: general organogram**.

**Figure 2:**
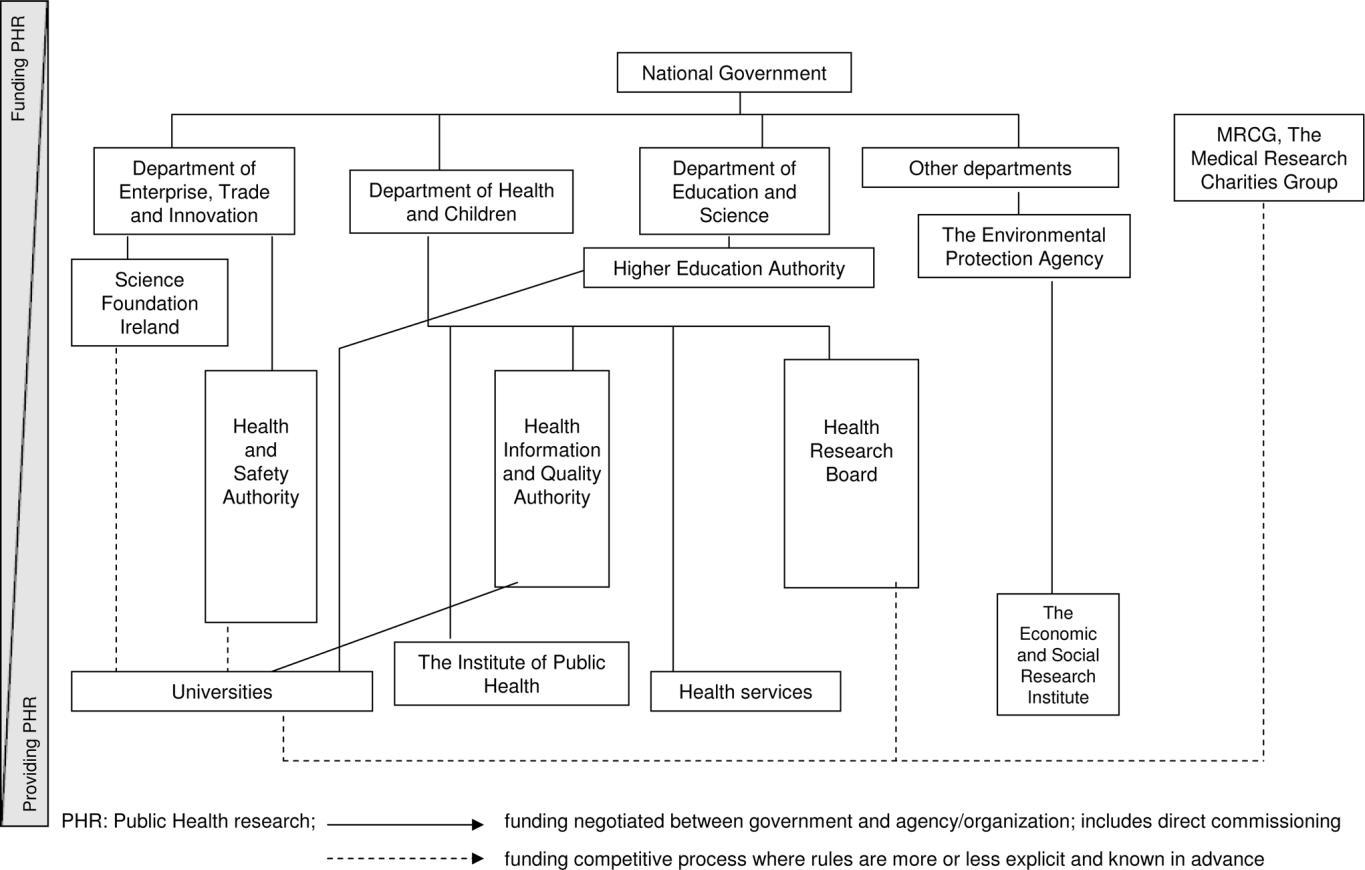
**Example of country specific organogram (Ireland)**.

For the sake of a balance between comprehensiveness and complexity, we chose to represent on organograms the organizations commissioning or performing public health research, linked by funding streams. A general diagram (Figure 1) was developed and a specific one was built for each country (and example in Figure 2). On each national organogram, boxes are given the different names of the national organizations and are linked by arrows: a full line for funding negotiated between government and agency/organization, including direct commissioning, and dotted line for funding through competitive processes where rules are more or less explicit and known in advance.

### Research Commissioners

The organisations at commissioning level which hold the budgets that fund public health research are ministries, national agencies and devolved (e.g. regional) organisations. The organizations identified in each country were described in relation to their mission, their web address, which organizations they fund, and if possible the budget allocated to research. The sources of funding and control included ministries of health, ministries of science, other ministries, directly from the central government or parliament, or from other sources. This section also identifies where devolved or regional funding agencies are involved in Public Health Research support. These are described as 'Regions', but in some countries they have substantial legislative and financial autonomy. Private not-for-profit organizations, at national level, for which the main source of financing was not public funds, are called 'Foundations' on the country profiles (and are called --charities in some countries).

The broad structures for science in EU countries have been described by the European Commission [8]. Most countries have a ministry with responsibility for science. In several countries the task is joined with education, including the tertiary (higher education) sector (Bulgaria, Ministry of Education, Youth and Science; Estonia, Ministry of Education and Research; Finland, Ministry of Education and Culture; France, Ministry of High Education and Research; Germany, Ministry of Education and Research; Italy, Ministry of Education, University and Research; Latvia, Ministry of Education and Science; Netherlands, Ministry of Education, Culture and Science; Portugal, Ministry of Science, Technology and Higher Education; Slovakia, Ministry of Education, Science, Research and Sport; Sweden, Ministry of Education and Research).

Increasingly now, however, countries have adopted the word 'innovation' in reflection of European concern for use research more as 'innovation' for economic and social ends [39] (Denmark, Ministry of Science, Technology and Innovation; Spain, Ministry of Science and Innovation). Moreover, some countries manage their science portfolios (and indeed formal representation at European level, for example in the European Science Foundations) through the business or economic ministry (Hungary, Ministry for National Economy; Ireland, Department of Enterprise, Trade and Innovation; United Kingdom, Department for Business, Innovation and Skills).

Ministries of science usually hold and allocate funds for science themselves, but in some countries allocations pass directly from the ministry of finance to research commissioning bodies, which may be independent or directly linked to government or parliament (Cyprus Research Promotion Foundation and Czech Science Foundation funded by governments; Hungarian Scientific Research Fund directly financed and accountable to parliament; Research Council of Lithuania, linked to the parliament).

Ministries of Health, either alone or in association with ministries of science, also fund health research agencies (ZonMw, the Netherlands Organization for Health research and Development; FAS, Swedish Council for Working Life and Social Research) or agencies that fund and perform research (Health Austria GmbH; INSERM, French National Institute of Health and Health Research; Health Research Board on Ireland; National Institute for Health Research, UK).

Research relevant to public health is also funded through different avenues of government, as well as in other policy areas and independent funds. In some countries, the parliament has set up research organisations directly. For example, the Hungarian Scientific Research Fund is directly accountable to parliament, while Sitra, the Finnish Innovation Fund, and the Vardal Foundation in Sweden are independent public funds under the supervision of the parliament. Devolved funding is found in Austria through the nine constituent federal states, in Belgium through two communities, in Germany through 16 states, in Spain through 17 autonomous regions, and in UK through administrations of Wales, Scotland and Northern Ireland. Ministries responsible for other policy areas such as food, environment, information technology and transport may also recognise health protection as an outcome of their policies, or scientists within their policy areas may link to medical and health issues.

Independent funding varies more by country. In some European countries, such as UK, fund-raising from the public for specific diseases has been directed towards research. Another model is also developing (such as in Spain), of not-for-profit foundations set up by industry: these are a tax-break for industry and are then able to undertake clinical research that is necessary for drug approval which should be independent of the manufacturer. Typically, these independent sources of finance have not been directed towards public health goals, although there are smaller independent foundations with areas of concern, such as for AIDS, or health systems research, or environmental concerns, where public health is significant partner.

### Mixed Organizations

A level of Mixed Organisations was created on the organogram to accommodate organizations that both allocate funds, sometimes with internal and competitive research, and also perform research (Health Austria GmbH, INSERM National Institute of Health and Health Research in France, Helmholtz Association of German Research Centres, Irish Health Research Board, Carlos III Institute in Spain, Medical Research Council in the United Kingdom). This structure also predominates for the Academies of Science in most of the former communist countries (Bulgaria, Czech Republic, Estonia, Hungary, Lithuania, Poland and Slovakia).

Another characteristic of this group of commissioners and performers of research is that they frequently are multi-site research organizations, with a considerable number of research groups and organizations involved in different financial and organisational arrangements. These research chains have a continuing link between the senior academicians who set the research agenda and the ministries who fund them. In the west European countries, there is some role for the ministry of health, although the main lines of the research are set by the research chain itself in discussion with the ministry of science. In the eastern academies, the autonomy is greater, although there have been mergers and restructuring in some countries as limited funds become competitive and moved towards universities.

### Research Performers

The organizations were grouped into four groups: State Institutes, Universities, Health Services and Independent Organizations (Figure 1). Information sought about each organization included its mission, web address, source of funding, relation towards specific ministries and areas of research. Often such information was too scattered and too poorly organised to be included in the text. However university and health services are always represented in the organograms in recognition of the importance of the research taken at these levels.

The State Institutes group includes organizations publicly funded, generally by a ministry, with different degrees of independence towards the government/ministry. The national structures with surveillance activity and the Schools of Public Health financed by the ministry of health were also included. The group of Independent organizations included fully private, private with some degrees of public funding, non-governmental organisations, and civil society organisations (usually not-for-profit).

The most visible national organizations undertaking public health research are national institutes primarily concerned with infectious disease and environmental control (Scientific Institute of Public Health, Belgium; National Institute of Public Health, Czech Republic; National Centre for Epidemiology, Hungary; ISS, National Institute of Health, Italy; InVS, Institute for Public Health Surveillance, France; RIVM, National Institute for Public Health & the Environment, the Netherlands; National Institute of Public Health, Poland; INSA, National Institute of Health, Portugal; Health Protection Agency, United Kingdom). In some countries, these institutes have broadened their roles to research on chronic disease control, epidemiology and health behaviours (National Institute for Health Development, Estonia; National Institute for Health and Welfare, Finland; Institute of Public Health, Ireland; National Institute of Public Health, Slovenia). These institutes usually decide their own research agenda, and funds are passed within their service budgets from the ministry of health. Institutes also have other ministries responsible for their financing (Dutch RIVM is co-financed by Ministry of Environment; Danish Institute is part of the university and is financed by Ministry of Science).

There are also a number of performers of public health research developing health interest within other disciplines: i) Institutes of environmental protection and occupational disease (Executive Environmental Agency, Bulgaria; Finnish Environment Institute; National Institute of Environmental Health, Hungary; National Research Centre for the Working Environment, Denmark; Finnish Institute of Occupational Health; Federal Institute for Occupational Safety and Health, Germany; Institute for Occupational Safety and Prevention, Italy; Institute of Occupational and Environmental Health, Poland); ii) Food safety (Federal Research Institute of Nutrition and Food/Max Rubner Institute, Germany; National Institute for Food and Nutrition Science, Hungary; Spanish Agency for Food Safety and Nutrition; National Food and Nutrition Institute, Poland); iii) Health services management, health economics and health technology assessment (Finnish Office for Health Technology Assessment; IRDES, Institute for Research and Documentation in Health Economics, France; Economic and Social Research Institute, Ireland; NIVEL, The Netherlands Institute for Health Services Research; Centre of Health Economics, Latvia).

Some countries in Europe maintain a National School of Public Health (Greece, funded by the Ministry of health; France, funded by ministries of health and science; Portugal, part of the university and funded by ministry of science). In most other countries, however, public health teaching and research has extended regionally within universities which have frequently gained equivalence with the original national school. At present, there is no full listing of university public health research departments.

### Academies

Significant historic structures in most countries of Central Europe have been the national Academies of Science. These were established mainly in the nineteenth century to protect and promote scientists (academicians) and have developed specific institutes across various science fields. The Academies retained important organisational links and power in the second half of the last century in the former communist countries, but have found less favour in the past two decades. They do not include public health sciences (usually an institute under the ministry of health) but usually do include institutes in allied fields (medicine, sociology). The Academies in some countries also retain important control of science commissioning, including the EU Framework programmes, and elsewhere the name 'Academy' remains for structures only for research commissioning or strategic advice (Table 3).

**Table 3:** Characteristics of Academies of Sciences

Country	Activities	Main funding	Description
Austrian Academy of Sciences http://www.oeaw.ac.at/english/home.html	Performer	Federal Ministry for Science and Research	Undertakes research in natural and technical sciences in institutes, research units and departments (commissions) across Austria. The Vienna Institute of Demography is part of the Academy.

The Bulgarian Academy of Sciences http://www.bas.bg/cgi-bin/e-cms/vis/vis.pl?s=001&p=0200	Mixed	State budget from National Assembly.	Has 77 institutes across sciences and humanities, and budget of €42.7 m (2009). There are 9 research fields (including 'Biomedicine and Quality of Life', 6 institutes, and 'Man and Society', 4 institutes), and funding from 89 FP7 projects. Also supports training for researchers and professionals, both independently and jointly with higher educational establishments. [The government plans to cut funds, create autonomous institutes and link them to universities.]

Academy of Sciences of the Czech Republic http://www.cas.cz/index.html	Commissioner and Mixed	State budget	Has 54 public research institutions and 7000 staff. It conducts basic research across the natural, technical and social sciences and the humanities. There is a Section of Biological and Medical Sciences and another on Bio-Ecological Sciences, Humanities and Social Sciences (that includes Social and Economic Sciences). It also forms the Grant Agency that distributes funds for open competitive grants, and manages the EU framework programmes.

Estonian Academy of Sciences http://www.akadeemia.ee/en/	Advisory	National government	An association for scientists in the formulation of science policy and foresight. There are 58 members and 19 foreign members, and an annual budget of €1.5 m from the national government.

Hungarian Academy of Sciences http://www2.mta.hu/?english	Mixed	National government	Has 47 separate research institutes - none are medical or public health. It has 11 programme Sections: one on Medical Sciences has five committees, with one of Preventive Medicine. HAS also supports joint research groups, including the Public health research group at the University of Debrecen.

Latvian Academy of Sciences http://www.lza.lv/index.php?mylang=english	Advisory/Commissioner	Parliament	An association of scientists functioning as an advisory body. From 2008, the Ministry of Education and Science of the Republic of Latvia has delegated the functions of the national funding agency for several European science programs and projects, and Latvian Academy scientists are national delegates in the EU research program committees and working groups.

Lithuanian Academy of Sciences http://lma.lt/index.php?lang=en	Advisory/ Mixed	Parliament	Has 5 advisory Divisions of Sciences, including Medical and Geosciences. It has four 'Associated Institutes', three within universities, including the Institute of Immunology in Vilnius.

Polish Academy of Science http://www.english.pan.pl/index.php?option=com_content&view=frontpage&Itemid=1	Mixed organizations	Ministry of Education and Science	A self-elected 'corporation of top scholars', with 76 research establishments managed from 6 Divisions (Medical Sciences division has 5 institutes, no public health), and 4000 researchers in 2005, including 'stations' in Brussels, Rome, Paris, Berlin and Moscow. It also distributes the EU Framework Research Programme funds.

Romanian Academy http://www.acad.ro/def2002eng.htm	Mixed	Ministry of Education, Research and Innovation	Covering sciences and humanities, with 183 members for life in 14 Sections, including Medical Sciences. There are 66 scientific (including social science) research units across the country. Scientists control their own research plans and in-house publishing of over 100 journals.

Slovak Academy of Science http://www.sav.sk/	Mixed/Commissioner	Ministry of Science - direct and competitive.	Has five divisions, 74 organisational units, a budget of €46.7 m and employs 3300 people. One division is medicine with 8 institutes, including oncology and heart disease, but no institute covers public health research. The Academy also manages the VEGA Grant Agency which in 2008 supported 1722 projects with €9.7 m.

Slovenia Academy of Arts and Sciences http://odmev.zrc-sazu.si/zrc/	Advisory		100 full and associate members are organised in 6 Sections, including Medical Sciences. There are 13 major independent institutes. The SAAS Scientific Research Centre coordinates 18 small institutes in humanities and social sciences, including the Socio-Medical Institute with a multidisciplinary approach.

### Research Strategies

We sought to identify health-research strategies or other statements of research priorities, to describe public health research in these strategies, and to analyse relationship with programmes and calls and other aspects of funding. We identified national research strategies in all countries, of which 17 explicitly referred to health and 11 to public health research themes (Table 4 and 5). Some research (and innovation) strategies are not formulated thematically but according to capacity building. In some countries, strategy is led by the governmental research agency rather than direct national research strategy. In Belgium research is not led at national level but by the communities. We found six research strategies directly for health and four referring to, or specifically for, public health. Some national health strategies, and public-health strategies, described aspects of research. For example, Finland has a public health programme "Health 2015" [40] that includes sections on public health research, including health promotion, health policy research and social epidemiology, and the UK has both a health research strategy and also a named public health research programme [41,42].

**Table 4:** Research strategies or statements of research priorities identified by STEPS in 2010 and reference to health and public health themes

Country	National research strategy/priorities			National health research strategy		Governmental health research agency strategy
		
	Strategy	Strategy referring to health	Strategy referring to public health research	Strategy	Strategy referring to public health research	
Austria	Y					
**Belgium**	Y					

**Bulgaria**	Y					

**Cyprus**	Y*					

**Czech Rep**.	Y	Y	Y	Y	Y	

**Denmark**	Y	Y	Y			Y

**Estonia**	Y					

**Finland**	Y	Y				

**France**	Y	Y	Y			Y

**Germany**	Y	Y		Y	Y	

**Greece**	Y	Y				

**Hungary**	Y					

**Ireland**	Y	Y	Y	Y	Y	Y

**Italy**	Y	Y		Y		

**Latvia**	Y	Y	Y	Y		

**Lithuania**	Y					

**Luxembourg**	Y					

**Malta**	Y					

**Netherlands**	Y	Y	Y	**	**	**

**Poland**	Y	Y	Y			

**Portugal**	Y*					

**Romania**	Y	Y				

**Slovakia**	Y	Y	Y			

**Slovenia**	Y	Y				

**Spain**	Y	Y	Y			

**Sweden**	Y	Y	Y			Y

**UK**	Y	Y	Y	Y	Y	Y

Y means that a strategy or mention of it could be identified; * Statements;  ** Included on the national strategy

**Table 5:** Research strategies or statements of research priorities identified by STEPS in 2010

Country	National research strategy	Health research strategy	Governmental health research' agencies strategies
**Austria**	The Austrian Council for Research and Technology Development Strategy 2020		

**Belgium**	FNRS Scientific research fund. Plan for harmonisation and action for research 2010 - 2014 (PHARE, Plan d'Harmonisation et d'Action pour la Recherche, Refinancement - Plan Stratégique du Fonds de la Recherche Scientifique - période 2010 à 2014. Avril 2009)		

**Bulgaria**	The National Strategy for Scientific Research, 2005 - 2013		

**Cyprus**	Statement in National Development Plan 2007-2013		

**Czech Rep**.	National Policy of Research, Development and Innovations in the CR for the years 2009 - 2015; National Research Programme	Departmental Programme of Research and Development of the Ministry of Health	

**Denmark**	Research 2015 - a basis for prioritisation of strategic research		National Research Centre for the Working Environment Research strategy

**Estonia**	Knowledge-Based Estonia II. Research, Development and Innovation Strategy for 2007-2013		

**Finland**	The Research and Innovation Council of Finland Research and Innovation Policy Guidelines for 2011-2015		

**France**	National Research and Innovation Strategy 2009		Inserm strategic plan (Notre Stratégie pour les Sciences de la vie et de la santé)

**Germany**	The High-Tech Strategy 2020	Roadmap for the German Health Research Program of the Federal Government, 2007	

**Greece**	Strategic Development Plan for Research, Technology and Innovation under the 2007-13 National Strategic Reference Programme		

**Hungary**	Government's Science, Technology and Innovation strategy 2007-2013		

**Ireland**	Strategy for Science, Technology and Innovation 2006-2013 Building Ireland's Smart Economy: a Framework for Sustainable Economic Renewal	Health Research Group Action Plan for Health Research 2009 - 13	Health Research Board Strategic Business Plan 2010 - 2014: The future of Irish health research

**Italy**	National programme of Research (Progamma Nazionale di Ricerca) 2010-2012	Strategies for the health research in the National Health System	

**Latvia**	Science in Latvia - National Research Programme 2009	National Research Programme Public Health	

**Lithuania**	Long-term science, research, and development strategy (until 2015)		

**Luxembourg**	National Research Fund CORE programme		

**Malta**	National Strategic Plan for Research and Innovation 2007-2010 - Building and sustaining the R&I enabling framework		

**Netherlands**	Science valued! - NWO (Netherlands Organisation for Scientific Research) Strategy 2007-2010		

**Poland**	National Framework Programme for research, 2005		

**Portugal**	Statement in "Commitment to science" (Compromisso com a ciência) 2006		

**Romania**	National strategy for research and development 2007 - 2013		

**Slovakia**	Long-term Plan of the State Science and Technology Policy by the Year 2015		

**Slovenia**	National research and development programme 2006 to 2010		

**Spain**	National Plan on Research, Innovation and Technology 2008-2011		

**Sweden**	The Swedish Research Council Research Strategy 2009-2012		FAS, Swedish Council for Working Life and Social Research strategic plan: "Research on people's working life, public health and welfare: Research strategy 2009-2012"

**U. Kingdom**	UK Science and Innovation Investment Framework 2004-2014	Department of Health's Science and Innovation Strategy Best research for best health; Scottish CSO's research strategy 'Investing in Research: Improving Health; The Northern Ireland 2007-2012 Strategic Plan 'Research for Health and Wellbeing'	Medical Research Council Strategic Plan 2009-2014 'Research changes lives'

### Programmes and calls

This section of the Country Profiles provided a place to describe identifiable national public health research, how it was supported and how much funding was available for each programme or call. It proved impossible to achieve this systematically because of the diversity of funding streams and organisations, as indicated above, and the lack of separation of public health from other related research - biomedical, clinical or wider determinants of health. In France, a specific organisation, Groupement d'Intérêt Scientifique - Institut de Recherche en Santé Publique (GIS-IReSP) has been established to monitor public health research activities, and to oversee funding (€2 m p.a.) from a range of institutions [43]. The UK research programmes directly related to public health (excluding health technology assessment) have defined budgets [41], which together amount to around €30 m annually, 4% of the total budget of the National Institute for Health Research.

Some research performers are funded directly, through regular negotiation with their commissioners - generally ministries. This generally applies to institutes of public health and in specialised fields such as environmental health. However, research is increasingly funded in response to competitive calls. Programmes and calls may be i) thematic, focused on a defined subject area; ii) general, such as to reinforce research capacity (scholarships, seminars, training, grants for visiting researchers); iii) open to any subject, with funding dependent on the quality of the proposal.

### European contacts

There is no organisation that presents all health research for Europe. The European Science Foundation, in Strasbourg, through its European Medical Research Councils, brings together both research commissioning and research performing organisations and assists thematic collaboration between 30 countries. However, its focus is on biomedicine [38] and it does not engage with ministries of health. Ministry of health international offices do not usually take a direct interest in health research, except when it impinges on national policy.

The European Commission has two structures that were included in the Country Profiles. The Directorate for Health has National Contact Points [44], usually but not necessarily in the ministry of health; and the Directorate for Research has National Focal Points [45], usually but not necessarily in the main research commissioning organisation, and (depending on the country size) subdivided into themes including health. Nevertheless, the prime concern of these contacts is to distribute information about European Commission activities relevant to member states, particularly funding programmes - that is, they distribute information downwards. Information passes upwards from countries to the European Commission formally through Advisory committees, but the work of these groups is rarely in the public domain.

## Discussion

Our study, related to the objectives of the World Health Organisation strategy Research for Health, has demonstrated the structures and variations in support for public health research in the 27 EU member states, based on the financial relationship between the organisations. More work is needed to determine actual financial flows and thematic programmes in member states. The European Union could contribute coordination of countries to achieve shared objectives in support of national public health policies and practice.

### Terminology

A critical component of studying public health research is the interpretation of words. Pang et al [3] propose that 'health research' involves many different types of research including biomedical, clinical, epidemiological, health systems and policies research, socioeconomic and behavioural research contributions, as well as on-going programme evaluations, surveillance and operational research activities embedded within health systems. It also includes research not usually considered to be health related -- for example, engineering studies to improve car or road safety or economic research leading to policy changes that affect poverty.

Our study on European countries describes 'public health' research systems as including all the areas in this list except biomedical research. Medicine extends from knowledge of the body (anatomy and physiology), its diseases (pathology), and its treatment (medicine, surgery, therapeutics, psychiatry), to understanding of normal and abnormal body function at cell and molecular levels, and at population-level developing epidemiology and sociology for population control of disease through environmental and behavioural change. Often when the term 'medical research' is used, it refers to laboratory and clinical research ('from lab to bedside'), particularly for pharmaceuticals research, without the broader public health concerns of medicine. 'Health services' and 'health care' are predominantly concerned with treatment and care of disease or injury, and the adjective 'health' is used instead of 'medical' partly to reflect the team of staff beyond medical doctors. Global health analysts have sought to distinguish the fields of operations research, implementation research, health systems research [46]. In Europe, these are considered as 'health services research' [47], and the broad term 'public health research' can embrace these as well as environmental, behavioural and policy research [25].

### Quality

The quality of information collected depends on both methods and responses. Ministries of health generally do not have a senior officer with adequate information about health research in their country. We have found the internet, with Google translate, to be a valuable method of exploring sources across a wide range of languages. We prepared initial descriptions based on these and the reports developed earlier in the *SPHERE *survey of ministries of health and of science. Subsequent comments and corrections were from (unpaid) informants across universities, research organisations and ministries of health. However, the data presented may represent an underestimate of practice, since we are not comparing clearly-defined organisational structures or categories. This should improve as mapping health research systems in Europe proceeds.

We could not identify the funds actually used for public health research. Chalmers has argued [48] that in the UK, three-quarters of public and charity (foundation) funds for medical research are directed to the laboratory and life sciences, and only 10% to clinical research (which is most relevant to the Cochrane Collaboration). Since then, the funding for clinical research has increased, but the allocation for public health research remains low [49]. In the USA in 2009, industry spent $75 bn (€53 bn) annually, compared with $47 bn (€33 bn) public spend and €18 bn (€13 bn) from other sources (including universities and foundations) [50], but again the allocation to the main public health research commissioning agencies is only 1-2% of this total [48]. In Switzerland, home to several global pharmaceutical companies, commercial funds are 90% of total national spending, and federal fund less than 1% of the total [51].

Work is needed to develop common typologies for research contracting and of the academic fields of public health research. Slovenia, for example, which manages all fields of research through a single agency [52], has developed an electronic database of commissioned research, but cannot identify public health research within this. The French GIS-IreSP has an important listing of public health research projects, although not classified by field [53]. The UK public sector together with the charities have developed a Health Research Classification System [54], and an analysis of non-commercial health' research by spend, using this classification, found that 70% was described as 'underpinning' and 'aetiology' (i.e. biomedical laboratory research), and less than 5% was on 'health services research' and just 2.5% on 'prevention' [55]. However, the classification has very limited detail in public health topics.

### Supporting health research

Pang et al [3] propose four functions and operational components of health research systems - stewardship (including vision and priorities), financing, creating and sustaining resources, and producing and using research. Mapping the national structures for health research is a step towards better research stewardship. It increases transparency, provides recognition of actors (including fund-raising, funding sources, production, translation/communication and advocacy), and facilitates collaborations in agenda setting. There is also growing interest in the evidence base needed for policy, which depends crucially on linking the policy interventions with funding of research on that policy (policy implementation research). Both the World Health Organisation [12] and the European Union [15] give international support to health research but it is their member states which are the major force, through their financial commitment and structures (commissioning and performing) to deliver the research.

In our previous EU-funded work *SPHERE*, responses from 20 of the 24 countries providing replies indicated that ministries of health, rather than ministries of science, were responsible for public health research [18]. We provided a broad definition of 'public health research', but it may be that some ministries of science gave a narrower interpretation of 'public health', based on infectious disease control and the traditional role of the institute of public health. However, while ministries of health recognised their interest in public health research, they were rarely able to identify a lead person directly responsible for public health research within the ministry. Sometimes, however, this may be more possible when dealing directly with policy areas: thus, for example, within a ministry, research may be supported by departments for fields such as patient safety, food, or sexually transmitted diseases, or for organisational issues such as primary care or cardiac services. But these were rarely brought together for a public health research strategy.

Our work for STEPS indicates movements and trends compared with data collected in *SPHERE*. There is a move towards engagement with of ministries of science/research. For example, in Spain, funding for the Institute Carlos III, a major 'mixed organisation' funder and performer of health research, has been transferred from the Ministry of Health to the Ministry of Science and Innovation. Second, there has been a merger of structures and efforts to improve coordination. Thus in Finland, KTL (National Public Health Institute) and STAKES (National Research and Development Center for Welfare and Health) have been merged to form the National Institute for Health and Welfare (THL) under the Ministry of Health. In France, GIS-IReSP, French Institute for Public Health Research (Groupement d'Intérêt Scientifique, Institut de Recherche en Santé Publique) results from merger of the group IReSP within the Institute of Public Health Research of the national research body INSERM. Third, there is increasing importance of research in universities, evolving from the historic separation of teaching in universities and research in the institutes. In Denmark, the National Institute of Public Health, previously under the Ministry of Health, is now under the Faculty of Health Sciences, University of Southern Denmark. Lastly, in Eastern European countries, the trend towards separating health institutes out of the Academy of Sciences continues (Bulgaria).

### European approach

The European Union is a relatively new organisation, and political power within Europe remains primarily at the level of member states. 'European research' is often considered as research funded by the European Union rather than research undertaken within a European country. A limiting factor has been language and nationalism. While most scientific public health research publications (and abstracting) are now in English [56,57], public health research is mainly undertaken in national languages, which have to be translated for science reporting. Moreover, commissioning of research through national funding agencies is often limited, by law or regulation, to research performers within the funding member state: cross-border health research is still rare. European collaborative projects, funded by the European Commission's Directorate for Research in the Framework Research Programme (FP7) have funded 70 collaborative public health projects over four years 2007-2010 [58] and the Directorate for Health's Health Programme has funded 300 projects (including networks, conferences and coordination) since 2003 in fields including environmental safety, health information, health determinants and health services [59]. But there is little integration between these programmes and national programmes, and the member states do not know what each other is doing in health research, although there are proposals for collaboration in funding for specific fields (Joint Programming).

While the majority of European member states have strategies for research, strategies for health research are less common. Member states have yet to develop research on public health systems. A team at GIS-IReSP, French Institute for Public Health Research, has created a database on research teams working in the field of public health, finding 313 teams within 238 groups in 2009 [60]. Reports by the Observatory on Health Research Systems (RAND Europe) for Spain [61], Sweden [62] and UK [63] primarily address biomedical research. COHRED supports health research at a global level for the 180 UN countries, but has not yet included EU countries as their focus is on low and middle income countries [64].

### Prospects

The European Region is the only WHO Region without a regional research policy and will wish to do more, both at member state level and collectively, to meet the challenge of the World Health Assembly's Research for Health Strategy. Coordination has been limited by problems in gaining participation and responses across all countries, especially as they require information to be provided and approved by ministries of health. Our approach using web-published sources as the basis for the reports gained information for all 27 EU countries, and we gained commentaries and corrections from national informants to achieve almost complete (96%) country coverage. The European Union has encouraged publishing governmental information on the internet, making retrieval more possible, and we recognise this may be a less feasible approach in low income countries.

To promote recording of country-level information on health research programmes by theme, we have now established a new collaboration, PHIRE (Public Health Innovation and Research in Europe), led by the European Public Health Association and co-funded by the European Commission's Directorate for Health [65]. We also contribute to an assessment of European research in a specific public health theme - food and health [66]. Nevertheless, the most important challenge is for ministries of health to recognise their prime importance for strategy and funding in public health research, and the value in coordination to achieve significant gains in knowledge development and translation. We suggest that the European Union's Committee of Ministers of Health should give greater attention to the Research for Health Strategy to which their governments have signed as members of the World Health Organisation.

## Competing interests

The authors declare that they have no competing interests.

## Authors' contributions

The study was conceived by MM, designed by MM and CC, undertaken by CC, analysed and written by CC and MM. All authors read and approved the final manuscript
